# Protocol for a randomized controlled trial comparing the Circle of Security-parenting (COS-P) with treatment as usual in child mental health services

**DOI:** 10.1371/journal.pone.0265676

**Published:** 2022-04-26

**Authors:** Aida Bikic, Johanne Smith-Nielsen, Søren Dalsgaard, James Swain, Peter Fonagy, James F. Leckman

**Affiliations:** 1 Child and Adolescent Mental Health Services Southern Jutland, Aabenraa, Region of Southern Denmark, Denmark; 2 Department of Regional Health Research, Faculty of Health, University of Southern Denmark, Odense C, Denmark; 3 Department of Psychology, Centre for Early Intervention and Family Studies, University of Copenhagen, Copenhagen, Denmark; 4 Department of Economics and Business, National Centre of Register-Based Research, Aarhus University, Aarhus, Denmark; 5 Institute of Clinical Medicine, University of Copenhagen, Copenhagen, Denmark; 6 Department of Child and Adolescent Psychiatry, Mental Health Services of the Capital Region, Glostrup, Denmark; 7 Department of Psychiatry and Behavioral Health, and Psychology, Stony Brook University, Stony Brook, NY, United States of America; 8 Department of Clinical, Educational and Health Psychology, University College London, London, United Kingdom; 9 Child Study Centre, Yale University School of Medicine, New Haven, Connecticut, United States of America; Prince Sattam Bin Abdulaziz University, College of Applied Medical Sciences, SAUDI ARABIA

## Abstract

**Background:**

The quality of a child’s attachment to its primary caregiver plays an important role for its long-term socioemotional development. While ‘secure’ attachment is associated with better outcomes, ‘insecure’ attachment is associated with a higher risk of externalizing and internalizing symptoms. Children referred to mental health services show much higher rates of insecure attachment than the general population, yet the parent-child relationship is rarely in treatment focus. Attachment quality is closely associated with parental sensitive responsiveness that is target of attachment-based interventions like Circle of Security (COS). COS has shown to improve attachment quality and the well-being of both children and parents. No randomized controlled trials have investigated the effect of COS on parental sensitivity and child psychiatric symptoms in child mental health services.

**Objectives:**

To investigate whether COS-Parenting (COS-P) can increase observed maternal sensitivity and decrease children’s psychiatric symptoms as an add on to treatment as usual (TAU).

**Methods:**

In a randomized controlled parallel superiority trial COS-P is compared with TAU for parents of children referred to child mental health services (n = 186). Families are randomized 2:1 to intervention or control group, if their child is between 3 and 8 years old and scores ≥ 93^d^ percentile on both the CBCL total score and the oppositional defiant disorder or conduct disorder subscale. Primary outcome is maternal sensitivity, secondary and exploratory outcomes include, among others, child psychiatric symptoms, parental stress and coping with children’s negative emotions. Outcomes and adverse events are assessed before (T0) and after 10 weeks of treatment (T1) and 6 months later (T2). Regression analysis and /or ANOVA will be used for all outcomes.

**Perspectives:**

Targeting the parent-child relation has the potential to reduce psychiatric symptoms in children. This trial will provide valuable information if attachment-based interventions like COS-P can enhance treatment as usual in child mental health services.

**Trail registration:**

ClinicalTrials.gov Identifier: NCT03578016.

## Introduction

Attachment is a neurobiologically determined instinct seen across almost all species, including humans, where it refers to children’s emotional bond with their primary caregiver(s). Infants are biologically predisposed to approach their parents for comfort and protection under distress and to use them as a ‘secure base’ from which to explore the world [1]. The quality of the child’s attachment is strongly associated with parental sensitivity [2] which is the ability to accurately perceive and interpret behavioral signals of the child and to respond to these signals in a prompt and adequate way [3]. Sensitive parents are emotionally available and responsive. Evidence shows that parental sensitivity is the strongest determinant of children’s secure attachment [4, 5]. Children of responsive and sensitive parents tend to develop secure attachment, feel free to play and express their positive and negative emotions and trust that their parents are available. On the other side, children of insensitive, unresponsive and inconsistent parents lack confidence that their parents are emotionally or physically available to them for comfort and protection and develop insecure attachment [2].

The quality of early attachment has been shown to play an important role in children’s long-term socioemotional development [6–8], including social competence and emotion regulation in childhood and adulthood [8, 9], and also psychopathology [6, 7, 10]. Evidence shows that early insecure attachment is associated with psychopathology in later life including emotional and conduct problems [11–13]. Several meta-analyses have shown that attachment insecurity predicts more externalizing and internalizing behavior problems in childhood and adolescence [6, 7]. The association between insecure attachment and externalizing symptoms is of moderate effect size (*d* = 0.58) [2]. Research also indicates that children referred to clinical psychiatric services tend to have more insecure attachment than the general population. This is in line with that insecure attachment is found to be a risk factor for psychopathology. While around 70% of non-referred children show secure attachment, is this only true for 20% of clinically referred children with early-onset conduct problems [14, 15]. Even lower percentages of secure attachment are found among children with ADHD [16, 17].

Disorganized attachment is the most dysfunctional insecure attachment pattern and it is thought to be caused by frightening and disconnected parental behavior [18, 19]. Longitudinal studies have linked disorganized attachment with hostility and hyperactivity, aggression and oppositional defiant disorder in children [20], with dissociative symptoms in 17- to 19-year-olds [21] and borderline personality symptoms at age 28 years. Accordingly, meta-analytic evidence shows that disorganization in early childhood is most strongly associated with later symptoms of psychopathology with the highest effect sizes for externalizing symptoms (*d* = 0.34 while it is less for internalizing symptoms, *d* = 0.15) [6, 7].

Compared to insecure attachments, early secure attachment relationships have been associated with better social competences [8]. Thus, evidence shows that the nature of a child’s attachment to their caregivers can have an important impact on the child’s emotional and social development and functioning both in childhood and later on.

Attachment-based interventions are designed to promote parental sensitivity, to change parental mental representations of their child, to improve understanding of the developmental needs of the child and to promote attachment security in the child [22]. A meta-analysis [23] of 70 studies targeting parental sensitivity, attachment in the child or both found that the most effective interventions had a focused, behavioral approach towards parental sensitivity. Additionally interventions enhancing parental sensitivity were significantly more effective in clinically referred families than other groups. Short term attachment approaches (under 16 sessions) targeting maternal sensitivity were found to be the most effective [23].

Sensitive parenting is not only important for infants and very young children. A longitudinal adoption study showed that continued high maternal sensitive responsiveness in both early childhood and adolescence was associated with the child’s continued secure attachment from age 1 to 14 years [24]. Further, mother’s sensitivity that changed from low in early childhood (age 1) to higher sensitivity in adolescence (age 14), corresponded to the child’s change from insecure attachment at age 1 to secure attachment in adolescence [24]. The study concluded that sensitive parenting in both early childhood and adolescence is important for the continuity of attachment across the first 14 years of life. Thus it is possible for parents to change their sensitivity level and herby affect a change in the child’s attachment even in adolescence.

### Circle of security

The Circle of Security (COS) model (http://www.circleofsecurity.net/) is a parenting intervention that leverages research on attachment relationships combining psycho-education with a mentalization-based approach [25, 26]. The main aim of COS is to promote parental insight into the child’s emotional needs and to enhance parental sensitivity towards these needs. Different versions of the COS program exist: The original version is called the COS-Intervention (COS-I) and it is an intensive 20-week group intervention, where the caregiver-child dyads are videotaped and analyzed individually several times during the intervention, and video-feedback is part of the program. The developers of the COS model, Cooper, Hoffman and Powell, have subsequently developed a shortened and condensed 8-week version of the program, called the COS-Parenting (COS-P) and this version is used in the present trial. Designed for scalability, the COS-P is less resource-intensive than most other attachment-based interventions [22] making it feasible to implement in a range of settings, for example in child psychiatric clinics, as a supplement to ongoing treatment. COS-P is a manualized intervention that uses pre-produced video clips of secure and challenging parent-child interactions, respectively. This material is used to initiate reflections in a group of parents. Participants are invited to reflect on their own relationship with their child and to discuss concrete examples on how they interact with their child. COS-P teaches the parents a specific language to help them verbalize feelings and behaviors. The aim is to help caregivers regulate their own cognitive, affective and behavioral responses to their child’s emotional needs; in other words, to maintain their capacity to mentalize and to respond in a sensitive manner even in difficult and challenging situations. The COS-P program has a friendly tone and uses non-judgmental language, and is easily accepted by parents and facilitators [27].

#### COS research

Although COS has been widely adopted internationally, there is only limited evidence on its effectiveness. Most studies to date have been uncontrolled, which also a meta-analysis [28] of 10 published and unpublished COS-I and COS-P studies concluded. Therefore, the meta-analysis could only calculate pre-post effects of treatment in the intervention group. Results indicate that COS improves secure attachment in the child as well as the quality of caregiving, while it reduces caregiver depression, all with moderate effect sizes. The largest effect size was seen for the improved caregiver self-efficacy [28].

#### COS-I

Most published research on COS is based on the more intensive 20 week COS-I version. COS-I has shown positive results in uncontrolled trials on both improving the attachment of infants [29, 30], toddlers and preschoolers [31] with a significant change in attachment from insecure to secure. A controlled Iranian trial of children age 4–6 years found significant improvements after the COS-I intervention on both the child’s attachment and child well-being after the intervention and in a 3 month follow-up, when compared with a control group [32]. This despite the small sample size (n = 48).

As described above, research suggests that insecure attachment may play a role in the development of (particularly) externalizing psychopathology. Therefore, it is a very relevant question, if the COS intervention could improve clinical symptoms in children, who are referred for psychiatric treatment. So far no randomized studies have examined the effects of COS-P on such symptoms, with the exception of an uncontrolled Australian study that investigated the effects of the 20 weeks COS-I [33]. In that study, families of children age 1–7 years (*N* = 83), who were referred to clinical services for behavioral problems and/or low levels of emotional well-being, were targeted. The intervention significantly improved children’s internalizing and externalizing symptoms, behavioral concerns and parental ratings of child protective factors defined as social/emotional resilience. Additionally, teachers reported significant reductions in the severity of externalizing symptoms [33]. The same study also showed significant reductions in self-reported levels of perceived stress and psychiatric symptoms in parents [34] and significant increase in caregiver reflective functioning, caregiving representations, and level of secure attachment in children [35]. Individuals with symptoms in the borderline/clinical range prior to the intervention, showed the greatest improvements. Due to the uncontrolled nature of the study, it is difficult to draw any firm conclusions about the effect of COS-I in this clinical population and further research is needed.

#### COS-P

Fewer studies have been published on the less intensive 8-week COS-P version. One randomized controlled trial in a study population of disadvantaged families with low income investigated the effect of COS-P on attachment and symptoms in children age 3–5 years (N = 141) [36]. No overall treatment effect was found for behavioral problems in children or attachment security. However, parents reported significant reductions in their use of unsupportive responses to child distress and improvement on one dimension of observed child executive functioning (greater inhibitory control) was also found. Further analyses indicated that maternal attachment style moderated the treatment effect on child attachment, i.e. children of mothers who scored high on attachment avoidance (intervention group) had higher rates of secure attachment and lower rates of disorganization post-intervention than children of mothers scoring high on attachment avoidance in the control group.

A small randomized and controlled trial investigated COS-P as an add-on to treatment as usual for parents of young children in the age range 0–4 years (*N* = 42) [37]. Target of the study were families referred to infant mental health clinics because of parenting relationship difficulties. There were no significant treatment effects on parents’ internal representations of the child and observed emotional availability during parent-child interactions, however, this may be due to a small sample size and lack of statistical power. Nevertheless a pre-post effect was observed in the treatment group, indicating that parents showed more balanced representations of their child and the proportion of emotionally available interactions significantly increased [37].

As this brief literature review shows, the evidence on both the COS-I and COS-P is limited, yet promising. Furthermore long-term effects of COS have not been evaluated as only one published study [32] had a follow up period. It is important to investigate long term effects of the intervention for several reasons: observable effects on parental sensitive responsiveness may take time to consolidate after the actual intervention period, but also to clarify if immediate effects of the intervention endure or are only of temporary nature.

To date, no randomized, controlled trials investigating the effect of COS-P on children with psychiatric symptoms have been published. Considering the impact of the quality of the parent-child relationship on the child’s wellbeing, it is essential to investigate, if specifically targeting the quality of the parent-child attachment relationship, by increasing parents’ relational capacity, could have a positive effect on reducing children’s current psychiatric symptoms. This is especially important as clinically referred children show a higher rate of insecure attachment than typically developing peers, indicating problems in the parent-child relationship.

### Hypothesis and aim of the study

We expect that an attachment based parenting intervention will benefit parents of children, who are referred to child psychiatric services and that the effect of the intervention also will extend to the child. We expect that the intervention will help parents to become more perceptive of the signals and needs of their child and improve parental sensitivity, parent-child relationship, children’s symptoms and parenting stress.

The aim of this trial is to investigate the effect of COS-P on maternal responsiveness and child psychiatric symptoms after the intervention and 6-months after the completion of COS-P. The primary objective is to investigate whether 10 weeks of COS-P have a positive effect on the observed sensitivity of mothers of children in the age range 3–8 years (age 2 years 11 months including), who are referred to psychiatric services with emotional and/or behavioral symptoms, all showing oppositional defiant symptoms.

The secondary objectives are to investigate whether COS-P has an effect on children’s psychiatric symptoms, children’s behavioral issues, maternal intrusiveness, parental coping with children’s negative emotions, parental reflective functioning and stress post-intervention and at 6 month follow-up.

An additional objective is to investigate possible moderators and predictors of treatment outcomes including parental attachment style, psychopathology, and depressive symptoms.

## Materials and methods

### Participants

Children in the age range 3–8 years (both inclusive), who were recently referred for assessment or are already in treatment for emotional and/ or behavioral symptoms at the Child Psychiatric Departments and their parents are invited. Only families fulfilling the following criteria can participate in the trial:

#### Inclusion criteria

Children, referred to mental health services who score ≥ the 93^d^ percentile on CBCL *total score* and *the ODD or conduct disorder subscale*; age between 3–8 years, both inclusive; informed consent from all legal guardians.

#### Exclusion criteria

**Children,** fulfilling any of the following exclusion criteria will not be included: autism spectrum disorder, serious psychopathology requiring immediate clinical attention (e.g. suicidality, psychotic symptoms); head injury or verified neurological disease; intelligence quotient (IQ) less than 80; a medical condition requiring treatment, and/or a lack of informed consent from legal guardians.

**Parents,** who have a known diagnosis of schizophrenia, known substance abuse and/or severe intellectual impairment.

#### Informed consent

Recruitment and information letters are handed out to parents of children in the target age group by their treating clinicians. Information folders are also visible in the waiting rooms of the clinics. Parents who show interest in the trial, will be offered an individual information meeting regarding trial details by a psychologist associated with the trial. After the meeting parents can decide if they wish to participate. Written consent of all legal guardians (later referred as parents) is required for the participation in the trial and will be collected by research staff. The trial will not intrude in any way with the child’s general assessment and treatment procedure.

### Procedures

Families, who provide written consent, are invited to fill out the Child Behavior Checklist for ages 1 ½- 5 or for ages 6–18 [38] and are screened for eligibility to participate in the trial. All children scoring above the cut-off total score for psychopathology which in a Danish sample is defined as a score ≥ 93^d^ percentile, *and* also a score ≥ the 93^d^ percentile on the oppositional defiant disorder (ODD) or conduct disorder subscale, are invited. These children can have a range of different clinical diagnoses and are thus included transdiagnostically. All eligible children are also assessed with the Reynolds Intellectual Assessment Scales (RIAS) [39] to ensure an IQ ≥ 80. Parts of the K-SADS interview, specifically the autism and psychosis part, are used to rule out autism and psychotic symptoms.

#### Control group

As we are interested in investigating the effect of COS-P as an add on to the usual treatment, both the intervention group and the control group will receive treatment as usual (TAU) at the clinic and/or in the community. TAU consists of clinical assessment and treatment and is not under the control of trial investigators. Clinical assessment might include intelligence tests, cognitive testing, school observations and parent and teacher questionnaires. TAU could involve psychoeducation, advising parents and in some cases medical treatment. Parallel to the trial, the participating children will undergo regular treatment procedures at the clinic and/or in the community. Possible medical treatment is decided by the parents and the treating specialist, who is unrelated to the trial. Families, who have children in medical treatment, are asked not to change the child’s medication dose during the ten weeks of the intervention. No other treatments are prohibited, but parents are encouraged not to participate in parent training treatments during the trial. Additionally we register all treatments that participants receive during the trial period, including TAU at all sites. Control group participants have the possibility to participate in the COS-P after 6 months in order to minimize attrition.

#### Intervention group

In addition to TAU that children in both groups receive, parents of children in the intervention group will participate in 10 manualized COS-P sessions. Although the original COS-P consists of 8 sessions, we choose to add two more sessions allowing more time for in depth discussion. Each of the sessions have a 2-hour duration and are conducted at the clinic by two COS-P-certified therapists. Treatment groups consist of 4–5 families. If parents miss a session, they are offered an 30-minutes make-up session.

#### Adherence

Therapists conducting the COS-P treatment are all certified in COS-P. To ensure adherence to the manual, therapists are required after each session to complete the COS-P session checklist (fidelity journal, unpublished manuscript). Additionally, therapists receive supervision from a supervisor appointed by the developers of the COS-P.

#### Randomization

In this parallel superiority randomized controlled trial participants are randomized 2:1 to the intervention group or control group in a web based randomization system. The allocation sequence is computer-generated with a varying block size kept unknown to the investigators. The allocation sequence has been set up by an independent statistician, who is not involved in the trial. Participants are screened for eligibility by research assistants and given an unique ID number. If all eligibility criteria are met, then the randomization is performed in a web based system and is stratified for site. Research staff has no access to the allocation sequence. Research assistants are informing families about their allocation and participation in the trial.

#### Trial sites

Participants are included at three sites, the Child Psychiatric Departments located in Aabenraa, Vejle and Odense.

#### Blinding

Due to the nature of the trial, it is not possible to blind the participants regarding group allocation. However, we will employ blinding in all other possible areas e.g. as outcome assessments and data analysis. Our primary outcome measure is a rating of video sequences of the parent-child interaction by blinded raters.

#### Data collection

All data is collected by research assistants, who are either clinical psychologists or psychology master students, with at least a bachelor degree in psychology. All research assistants are trained on the different outcome measures, that are collected. Data consists of video recordings of different parent-child interactions, video recordings of a child interview and questionnaires filled out by the parents about the child and themselves. Data is collected at the time points below.

### Measures

#### Time points

Assessments are collected at three time points:

T0: before intervention; T1: within 2 weeks of the completion of the intervention and T2: 6 months after the completion of the intervention.

**Fig 1:**
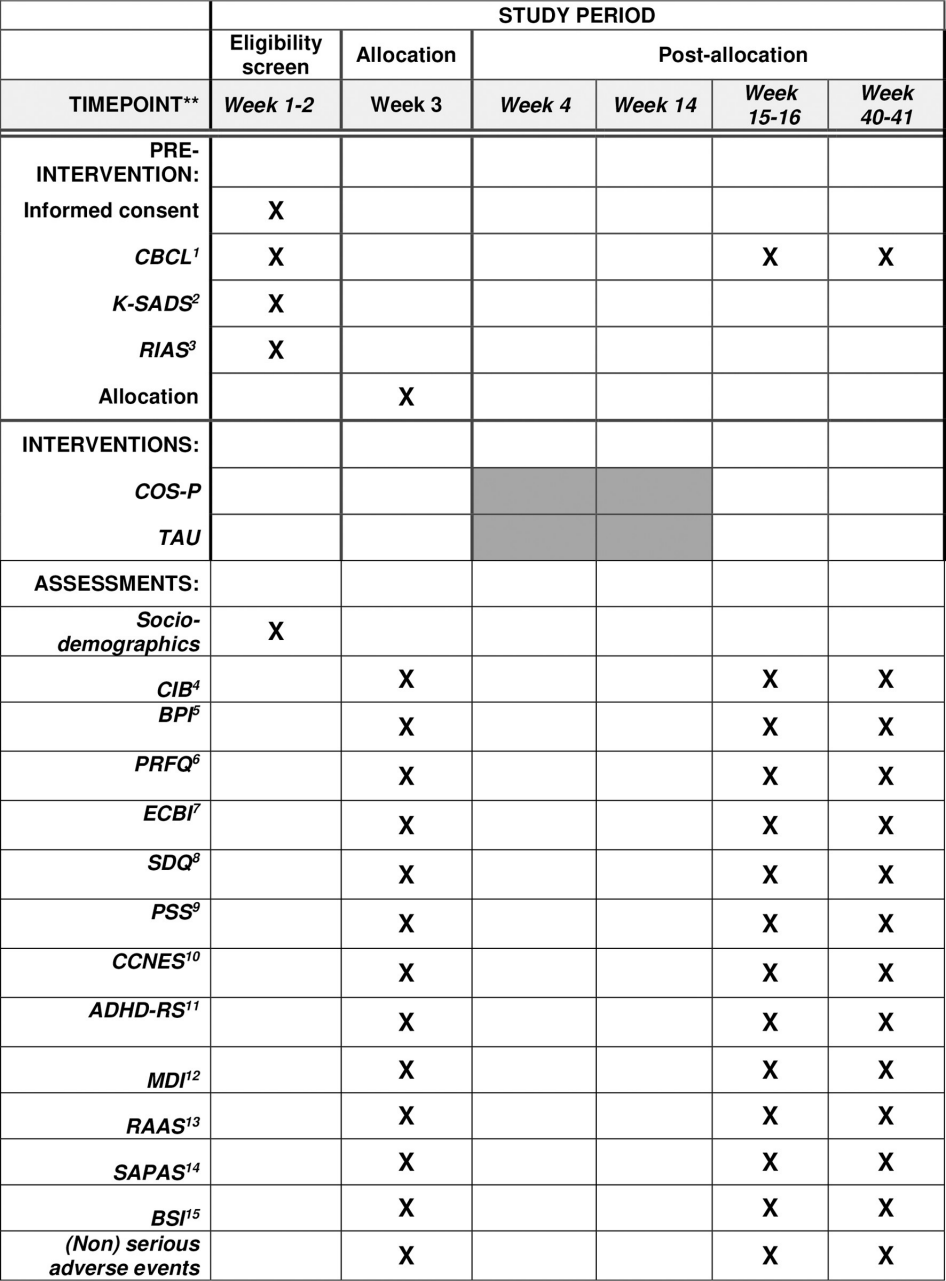
SPIRIT schedule of enrolment, interventions, and assessments. 1)CBCL-Child Behavior Checklist; 2) K-SADS-Kiddie-Schedule for Affective Disorders and Schizophrenia- autism and schizophrenia part; 3) RIAS-Reynolds Intellectual Assessment Scales; 4) CIB-Coding interactive behavior; 5) BPI-Berkeley Puppet interview; 6) PRFQ-Parental Reflective Functioning Questionnaire (; 7) ECBI-Parent-rated Eyberg Child Behavior Inventory; 8) SDQ-Parent-rated Strengths and Difficulties Questionnaire; 9) PSS-Parental Stress Scale; 10) CCNES-Coping with Children’s Negative Emotions; 11) ADHD-RS—Attention Deficit Hyperactivity Disorder-Rating Scale; 12) MDI-Major Depression Inventory; 13) RAAS -Revised Adult Attachment Scale; 14) SAPAS-Standardized Assessment of Personality-Abbreviated Scale; 15) BSI-Brief Symptom Inventory.

An overview of all assessment points and outcomes can be found in Fig 1.

The outcome measures described below are used to collect data on the child and the parents. All measures have shown satisfying psychometric properties including validity and reliability ratings. Video recordings of parent-child interactions will be rated by reliable and blinded raters. Inter-rater agreement will be calculated on a random subset of video recordings (20%).

#### Parent-child interactions, child behavioral and emotional functioning

**Coding interactive behavior (CIB)** is a clinician-rated measure coding adult-child interactions applicable for children from the newborn stage and up to adolescence [40]. In the current trial we are using the preschooler version. Video sequences of several parent child interactions are observed and coded on a 5-point Likert scale by a blinded rater, where 1 implies a minimal level of a specific behavior or attitude and 5 implies the maximal level. The parent child interactions are filmed during free play, structured play (puzzle) and a child-frustrating task. Coding of these situations is focused on the global nature, flow of the session and the interactive involvement of child and parent.

The coding scheme for the preschooler version consists of 44 scales; 22 scales regarding the adult behavior (like e.g. acknowledging, intrusiveness-overriding, hostility, praising) 15 scales regarding the child behavior (like e.g. child positive affect, withdrawal and compliance to parent), and 7 dyadic scales regarding the interaction between child and parent (like e.g. dyadic reciprocity, adaptation-regulation). These different scales are condensed into eight composite scores regarding the a) *parent*: 1) sensitivity, 2) intrusiveness and 3) limit setting; b) *child*: 4) involvement, 5) withdrawal and 6) compliance and c) *the dyad*: 7) dyadic reciprocity and 8) dyadic negative states. The CIB has shown good psychometric properties [40]. CIB has been used in a number of studies and has shown adequate construct and predictive validity, as well as test–retest reliability [41–44].

**Child Behavior Check List (CBCL) or CBCL/11/5-5** is also called the *Achenbach System of Empirically Based Assessment (ASEBA)* [45]. The CBCL is a parent-reported questionnaire screening for emotional, behavioral, and social problems of the child. Two different versions of the CBCL exist depending of the age of the child: a) the preschool version (age 1,5–5 years); and b) the school -aged child version (age 6–16 years). The CBCL has a total score and two indexes: *internalizing and externalizing* symptoms. The CBCL also contains specific subscales associated with disorders from the *Diagnostic and Statistical Manual of Mental Disorders* [46]: anxiety, oppositional defiant disorder, conduct problems, somatic problems, affective problems, and attention deficit disorder. A high rate of reliability between the scales of the CBCL and actual psychiatric diagnoses has been shown [47].

**Berkeley Puppet interview (BPI)** [48, 49] is a semi-structured interactive interview for children. The interviewer uses two hand puppets providing contradictory statements (“I’m a happy child” and “I’m not a happy child” “What about you?”) to gain access to the children’s perspective regarding their own emotional and behavioral problems, peer acceptance, social functioning and parent-child relationship. The child interview is videotaped and rated by blinded raters. The BPI has shown acceptable psychometric properties [49, 50].

**Parent-rated Eyberg Child Behavior Inventory (ECBI)** [51] is a 36-item parent-reported questionnaire about the behavior of their child (age 2–16 years). Each question asks how frequently a specific behavior occurs *and* if the behavior is a problem for the parent. ECBI is both used in clinical practice and intervention studies and contains two subscales: 1) the *Intensity subscale* measures how frequently each behavior occurs rated on a 7-point Likert-type scale and 2) the *Problem subscale* contains all the previously rated questions, but it is rated as a yes–no response about the parent considering the child’s behavior to be problematic. ECBI has good internal consistency, with alpha values for the Intensity and Problem Scale 0.93 and 0.91 [52], respectively and it is sensitive to detect intervention effects [53].

**Parent-rated Strengths and Difficulties Questionnaire (SDQ)** [54] is a 25-item questionnaire about strengths and weaknesses of the child. Additionally there is one impact question about whether the parent thinks that the child has a problem. If that is the case, then chronicity, distress, social impairment, and burden to others are also addressed. SDQ results in a total difficulties score, including four subscales regarding emotional symptoms, conduct problems, hyperactivity, and peer problems. Additionally, there is a prosocial behavior subscale. The Danish version of the SDQ has been found to have acceptable psychometric properties [55, 56].

**Attention Deficit Hyperactivity Disorder-Rating Scale (ADHD-RS) or Pre-school ADHD-RS** [57] is a parent rating scale of ADHD symptoms. The Danish version consists of 26 questions, including oppositional behavior questions, all rated on a 3-point Likert scale. ADHD-RS consists of 3 subscales: inattention, hyperactivity and oppositional behavior subscale [58]. ADHD-RS has shown to be valid and clinically feasible in a Danish population [57].

#### Parents reflective functioning and parenting experiences

The following questionnaires are self-reports of parents about their own perceptions and symptoms:

**The Coping with Children’s Negative Emotions Scale (CCNES)** [59] is a self-report measure for parents on how they would likely respond in 12 imaginary situations, in which their child is distressed. For example “If my child becomes angry because he/she is sick or hurt and can’t go to his/her friend’s birthday party, I would..” and the parent can then choose between 6 options for each of the 12 questions. Questions are rated on a 7-point Likert scale from 1 = very unlikely to 7 = very likely. Coping with Children’s negative emotions is a valid and reliable measure [59] and it results in six parent reaction patterns: 1) emotion focused, 2) problem-focused, 3) minimization, 4) punitive, 5) expressive encouragement, and 6) distress coping reaction pattern. CCNES has shown good reliability and validity [59].

**Parental Reflective Functioning Questionnaire (PRFQ)** (short 18 item version) measures levels of parental mentalization understood as the capacity of the parent to reflect on and understand the mental states and internal motivation of one’s self and their child. Mentalization is about being able to understand that feelings, beliefs and goals play an essential role in the well-being and development of the child. PRFQ consists of 18 items and results in three categories:1) Pre-Mentalizing modes 2) Certainty about mental states and 3) Interest and curiosity about mental states. The PRFQ has shown preliminary satisfactory validity and reliability [60–62].

**Parental Stress Scale (PSS)** [63] is measuring the level of stress the parents experience in raising a child. The PSS consists of 18 items depicting positive and negative themes of parenthood. The items are rated on a 5-point Likert scale ranging from 1 =“strongly disagree” to 5 =“strongly agree” and result in an overall score, that can range between 18–90. Higher scores indicate greater stress. PSS has satisfactory levels of internal reliability (0.83), and test-retest reliability (0.81) [63].

#### Parental psychopathology and attachment

In order to investigate possible mediators and moderators of treatment, we are also measuring symptoms and attachment quality of the parents with the following scales:

**Standardized Assessment of Personality-Abbreviated Scale, (SAPAS)** [64] is an screening questionnaire for personality disorder/ dysfunction with only 8 yes/ no questions. Although the scale is very brief, a score of 3 positive responses or more is associated with a personality disorder. SAPAS can correctly identify 90% of patients with a DSM-IV personality disorder. Its sensitivity (0.94) and specificity (0.85) are satisfactory [64].

**Brief Symptom Inventory (BSI)** Is a short 18-item version of the SCL-90-R instrument Symptom Checklist-90 (SCL-90) [65]. The BSI-18 [66] screens for psychiatric disorders and psychological distress and has three 6-item subscales: somatization, depression, and anxiety. The BSI has shown to be a reliable instrument for the assessment of psychological distress with good psychometric properties [67].

**Major Depression Inventory (MDI)** [67] is a 12-item self-report depression questionnaire developed by the World Health Organization. It is used to estimate depression symptom severity in the last 2 weeks and is based both on the DSM-IV symptoms of major depression and the ICD-10 symptoms of moderate to severe depression. The MDI has shown acceptable psychometric properties [68].

**Revised Adult Attachment Scale (RAAS)** [69, 70] is an 18-item scale assessing three adult attachment dimensions: 1) *Close*: comfort with intimacy, 2) *Depend*: ability to depend on others and 3) *Anxiety*: about being rejected or unloved. It is rated on a 5-point Likert scale ranging from 1 (not at all characteristic of me) to 5 (very characteristic of me). In a sample of undergraduates, Cronbach’s alphas for the close, depend, and anxiety subscales were .77, .78, and .85, respectively [69].

**Fig 2:**
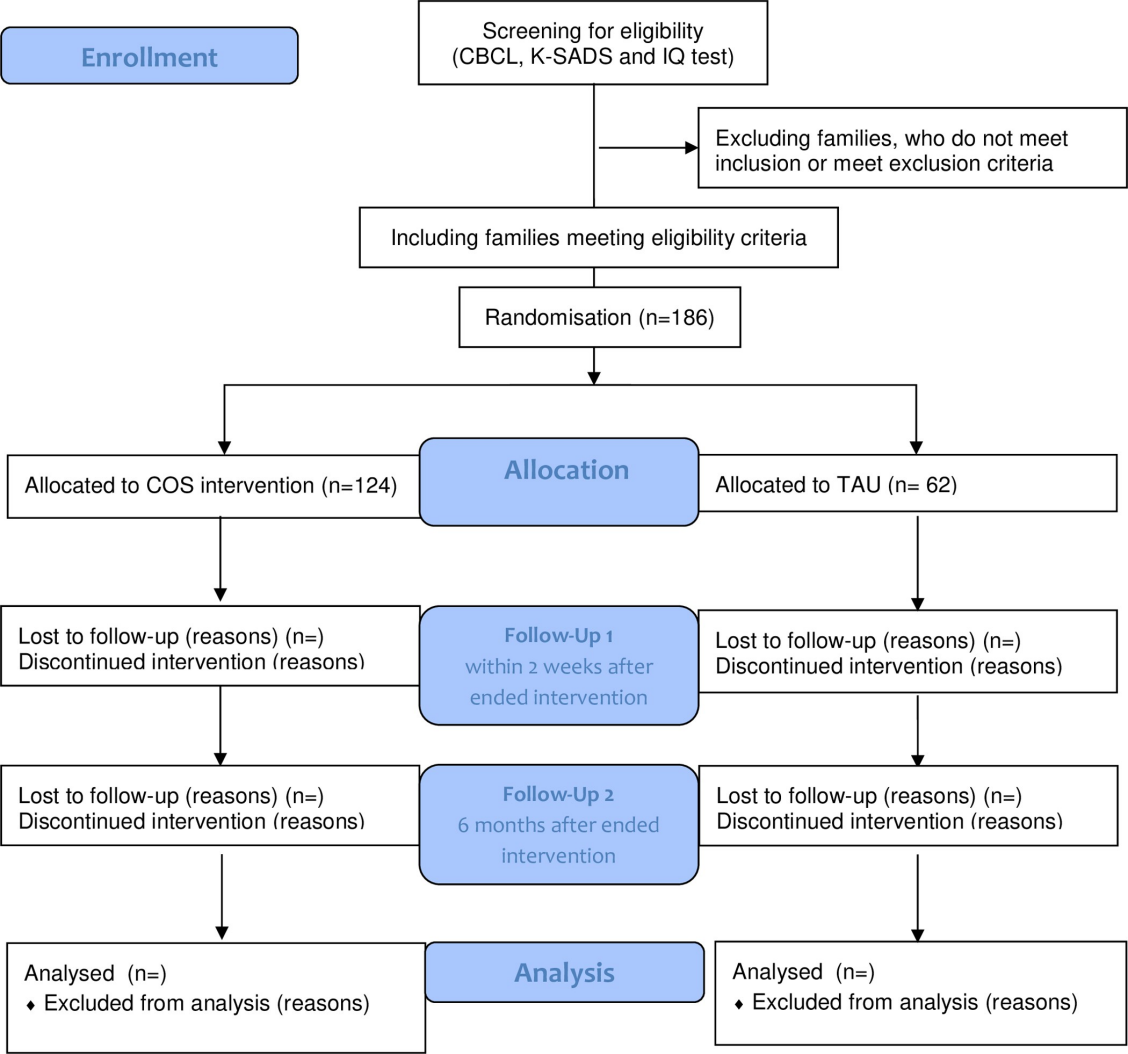
CONSORT flow diagram. Flowchart of the different phases of the COS-P trial.

An overview of the progress through the different phases of the current trial can be found in Fig 2.

### Outcomes

#### Primary outcome

The primary outcome in our trial is parental sensitivity of the mother (or primary caretaker) as measured with the Coding Interactive Behavior (CIB). Parental sensitivity is a composite score consisting of the following subscales: Acknowledging, Elaborating, Parent Gaze/Joint Attention, Positive Affect, Vocal Appropriateness, Appropriate Range of Affect, Resourcefulness, Praising, Affectionate Touch, Parent Supportive Presence.

#### Secondary outcome measures

All secondary outcomes are measured at baseline, at follow-up after the intervention period, and at 6 months follow up.

1. Maternal Intrusiveness: Measured by observing mother-child interactions and is rated using "Coding Interactive Behavior" (CIB)
2. Limit Setting (CIB)
3. Dyadic reciprocity (CIB)
4. Negative states (CIB)
5. Child Behaviour Check List (CBCL)-externalized symptoms
6. CBCL-internalized symptoms
7. Eyberg Child Behaviour Inventory (ECBI) Parent questionnaire on child behaviour
8. Berkeley Puppet Child interview
9. Coping with children’s negative emotions scale
10. Parenting Stress Scale
11. SDQ

### Exploratory outcomes

1. Parental Reflective Functioning Questionnaire
2. ADHD-RS: Parent questionnaire on child ADHD symptoms
3. Coding Interactive Behavior
4. Child Behaviour Check List
5. Major Depression Inventory: Parent questionnaire on their own depressive symptoms
6. Revised Adult Attachment Scale (RAAS): Questionnaire regarding the attachment style of the parent at baseline
7. Standardized Assessment of Personality (SAPAS) -parent: at baseline
8. Brief Symptom Inventory (BSI)

### Risks and side effects

The COS-P intervention has been used extensively in Europe, USA and Australia and translated into eight languages. No associated risks have been reported in previous trials and the intervention is not assumed to have negative effects. Patients’ TAU will not be affected by the participation in this trial.

The participating children, who are on medication are required to be on a 4 weeks stable medical treatment. Families will be asked not to initiate or change the child’s medication during the trial period of 10 weeks, Nevertheless, if the change in medication is necessary, the final decision will be made by parents and the treating physician, who is unrelated to the trial. Possible changes in medication status during the trial will be registered at all assessment points.

### Discontinuation and withdrawal

Enrolled families can withdraw from the trial without further explanation at any point. If a child or a parent requires more intensive care as inpatient hospitalization, the participant can be exited from the trial.

To promote retention and completion of follow-up, children will receive gift cards worth total of 400 DKK. If participants decide to leave the trial earlier, they will receive gift cards for the attended assessment sessions. Transportation costs will be reimbursed for all meetings.

## Statistical plan and data analysis

### Sample size and power analysis

We are planning a trial of a continuous response variable Coding Interactive Behavior from independent control and experimental participants allocated at a 2:1 ratio.

Based on a meta-analysis of attachment-based interventions [71] we expect a standardized effect size of *d* = 0.44 for comparing the change in maternal sensitivity from baseline to follow-up between the intervention group and the control group. Using significance level of 5% and power of 80%, and with a 2:1 randomization ratio, 124 participants in the intervention group and 62 in the control group are needed (total *N* = 186).

### Analytical model

For the intent to treat analysis of the primary, secondary and explorative outcomes, regression analysis and /or ANOVA will be used. For not normally distributed data, robust standard errors, truncation or transformation will be used. For missing data, the direct maximum likelihood method (full information likelihood) or multiple imputations will be used. In addition, per protocol analyses will be conducted for patients completing at least seven sessions, using regression analysis and /or ANOVA with robust standard errors, truncation or transformation when needed.

## Discussion

Considering the meta-analytic evidence on associations between the quality of attachment, psychopathology and the social functioning of the child, it is important to investigate if an attachment-based intervention could enrich the current child psychiatric treatment. Research is emphasizing the essential impact of the quality of the parent-child relationship on the child’s wellbeing. Therefore it is important to target parental sensitivity in interventions and to investigate its impact on the psychiatric symptoms of the child. Today, there is a general lack of interventions aiming to improve parental sensitivity and attachment security in populations of children referred to treatment in child psychiatry. This randomized, controlled trial with clinically referred children to mental health services will produce new and important knowledge regarding if parent training can improve parental sensitivity and children’s psychiatric symptoms. It is essential to investigate if child psychiatric treatment can benefit from interventions targeting the parent-child relationship. Ideally, children referred to psychiatric services and their parents may benefit from the intervention by improving parental sensitivity and decreasing the symptom load of the child as well as reducing parental stress. Helping parents to become more responsive to their children’s emotional needs and improving their relational capacity, could possibly impact on their children’s current psychiatric symptoms. Especially young children with emotional and behavioral symptoms are often not offered treatment within the current Danish psychiatric system because of their unspecific symptoms. If the intervention shows significant positive results, the COS-P could be easily implemented both at the psychiatric child clinics and in community settings. This would make it possible for many families to access help at an early stage and potentially prevent the development of more severe psychopathology in children, thereby improving their overall mental health and reducing adverse outcomes associated with mental health problems.

### Trial status

Trial registration: ClinicalTrials.gov Identifier, registered on July 5, 2018.

Protocol version 3, date 11.11.2020. The first participant was enrolled August 15th, 2018. Recruitment is currently ongoing till 2023. Currently 64 participants have been randomized. Recruitment of the trial has been impacted by the ongoing Covid-19 pandemic, slowing down recruitment as group interventions were not allowed for a longer period of time. Additional details about the trial registration are presented in Table 1.

**Table 1:** Trial registration data set.

Data category	Information^32^
Primary registry and trial identifying number	ClinicalTrials.gov
	NCT03578016
Date of registration in primary registry	July 5th, 2018
Secondary identifying numbers	17/7474; S-20170032
Source(s) of monetary or material support	Psychiatric Research Foundation in Region of Southern Denmark, A.P. Møller Foundation, Jascha Foundation
Primary sponsor	Psychiatric Research Foundation in Region of Southern Denmark
Secondary sponsor(s)	Jascha Foundation, A.P. Møller Foundation
Contact for public queries	Aida Bikic e-mail: abikic@health.sdu.dk
Contact for scientific queries	Aida Bikic e-mail: abikic@health.sdu.dk
	University of Southern Denmark, Odense, Denmark
Scientific title	Protocol for a randomized controlled trial comparing the Circle of Security-parenting (COS-P) with treatment as usual in child mental health services
Countries of recruitment	Denmark
Health condition(s) or problem(s) studied	Oppositional behavior, child psychiatric symptoms
Intervention(s)	Active comparator: Circle of Security-Parenting (COS-P), a group treatment for families
	Control group: Treatment as usual (TAU)
Key inclusion and exclusion criteria	Ages eligible for study: 3–8 years
	Sexes eligible for study: both
	Accepts healthy volunteers: no
	Inclusion criteria: Children, referred to mental health services who score ≥ the 93d percentile on CBCL total score and the ODD or conduct disorder subscale; age between 3–8 years, both inclusive; informed consent from all legal guardians
	Exclusion criteria: *Children*: autism spectrum disorder, serious psychopathology requiring immediate clinical attention (e.g. suicidality, psychotic symptoms); head injury or verified neurological disease; intelligence quotient (IQ) less than 80; a medical condition requiring treatment, and/or a lack of informed consent from their custodians.
	*Parents*: a known diagnosis of schizophrenia, known substance abuse and/or severe intellectual impairment.
Study type	Interventional
	Allocation: randomized intervention model. 2:1 assignment masking: investigator and outcomes assessor are blinded.
	Primary purpose: treatment
	Phase III
Date of first enrolment	August 15th, 2018.
Estimated primary completion date	December 2023
Estimated study completion date	2024
Target sample size	186
Recruitment status	Recruiting
Primary outcome(s)	Coding Interactive Behavior (CIB): parental sensitivity
Key secondary outcomes	1. Maternal Intrusiveness: (CIB)
	2. Limit Setting (CIB)
	3. Dyadic reciprocity (CIB)
	4. Negative states (CIB)
	5. Child Behaviour Check List (CBCL)-externalized
	6. CBCL-: internalized symptoms
	7. Eyberg Child Behaviour Inventory (ECBI)
	8. Berkeley Puppet Child interview
	9. Coping with children’s negative emotions scale
	10. Parenting Stress Scale
	11. SDQ

## Supporting information

S1 Checklist SPIRIT 2013 checklist: Recommended items to address in a clinical trial protocol and related documents*. (DOC)
